# Effects of mind-body exercise on individuals with ADHD: a systematic review and meta-analysis

**DOI:** 10.3389/fpsyt.2024.1490708

**Published:** 2024-12-09

**Authors:** Jin Peng, Weiran Wang, Yiting Wang, Fengting Hu, Mingyuan Jia

**Affiliations:** ^1^ Department of Physical Education, Dong-A University, Busan, Republic of Korea; ^2^ Department of Physical Education, College of Art and Physical Education, Hanyang University, Seoul, Republic of Korea

**Keywords:** mind-body exercise, ADHD, attention, adjunctive therapy, quantitative analysis

## Abstract

**Objective:**

To explore the effects of mind-body exercise (MBE) on ADHD through a systematic review and meta-analysis.

**Methods:**

After identifying relevant search keywords based on the study’s technical terminology, research articles were retrieved from five databases. Two researchers independently screened the results to select studies that met the inclusion criteria. A random-effects model was used to conduct a meta-analysis on the included studies.

**Results:**

The findings indicate that MBE interventions significantly improved attention in individuals with ADHD [SMD=-0.97, 95% CI (-1.56, -0.39), P < 0.05]. However, the meta-analysis found no evidence that MBE improved executive function, emotional issues, or hyperactivity/impulsivity in ADHD patients.

**Conclusions:**

MBE is beneficial for attention improvement in ADHD patients. However, further evidence is needed to support its efficacy as an adjunctive treatment for other symptoms.

**Systematic review registration:**

https://www.crd.york.ac.uk/prospero, identifier CRD42023447510.

## 1 Introduction

Attention-Deficit/Hyperactivity Disorder (ADHD) is a neurobehavioral condition that affects the development of children and adolescents, often continuing to impact individuals throughout their lives. Although commonly diagnosed during school years, ADHD has a lifelong impact (1, 2). Globally, epidemiological studies estimate that approximately 5.3% of children and adolescents are affected by this disorder (3), with the prevalence in adults reaching around 2.8% (4). Children with ADHD typically exhibit symptoms such as inattention, lack of self-control, and impulsive behavior (5). Epidemiological evidence also suggests that a significant portion of adults are affected by this disorder (6). ADHD can occur across all age groups (7, 8), imposing considerable burdens and negative impacts on individuals, families, and society at large, such as lower educational attainment (9), family conflicts (10), and increased rates of criminal behavior (11). Additionally, ADHD is associated with other neurodevelopmental disorders (12, 13).

Given these challenges, it is crucial to find effective treatments for individuals with ADHD. Psychostimulant medications are commonly used as the first-line treatment (14). However, these medications may produce side effects such as headaches, insomnia, weight loss, and seizures (15, 16). Over the past two decades, concerns about the adverse effects and negative impacts of pharmacological treatments (17) have spurred the development and application of non-pharmacological interventions for ADHD. Various interventions, including physical activity, neurofeedback training, and cognitive interventions, have emerged as complementary treatment options. These methods offer advantages such as lower costs, simpler implementation, and potential additional benefits.

Exercise, in particular, is often viewed as a low-cost and safe adjunctive treatment option (18). Mind-body exercises (MBE), such as yoga, dance, Pilates, and Tai Chi, have garnered significant attention for their effectiveness in improving mental health conditions, emotional problems, and related disorders (19). MBE involves a variety of movements, such as the rapid transitions in dance or the dynamic stretching in yoga (20). Studies have shown that interventions involving yoga, Pilates, and dance can significantly improve attention deficits (21, 22), cognitive performance (23), and sleep quality (24). The meditative component of MBE helps regulate attention and awareness (25), which may directly address the inattention symptoms characteristic of ADHD. Overall, the unique movement patterns of MBE have demonstrated positive effects in managing certain neurological and psychological disorders, suggesting that MBE could be an effective treatment option for ADHD (26).

Although several meta-analyses have examined the impact of MBE on various disorders, there is currently no systematic review or meta-analysis specifically exploring the effects of MBE on ADHD. Consequently, it remains unclear whether MBE can be considered an effective treatment for individuals with ADHD. This study aims to investigate the impact of MBE on ADHD through a meta-analysis, providing preliminary evidence and relevant insights for the clinical management of this disorder.

## 2 Materials and methods

This study was conducted in accordance with the guidelines outlined in the Cochrane Handbook for Systematic Reviews of Interventions and has been registered with PROSPERO (CRD 42023447510).

### 2.1 Search strategy

To accurately identify relevant studies, we initially conducted a preliminary search to determine precise keywords and content by examining the keywords and citation lists of relevant literature. As of June 2024, a comprehensive search of five databases (PubMed, EMBASE, the Cochrane Central Register of Controlled Trials, Web of Science, and Scopus) was conducted to identify pertinent studies. Detailed search terms and strategies are provided in **Supplementary Material 1**.

### 2.2 Inclusion and exclusion criteria

1. Studies included must involve participants diagnosed with ADHD using standardized diagnostic criteria such as DSM-IV or DSM- V.
2. Only randomized controlled trials (RCTs) were included. Observational studies, cross-sectional studies, animal studies, and other non-randomized controlled trials were excluded.
3. The experimental group must have received MBE interventions, while the control group either did not receive this intervention or received no intervention at all.
4. Studies must report outcomes relevant to the focus of this research, such as attention, emotional regulation, executive function, hyperactivity/impulsivity, with clear pre- and post-intervention data. Studies that did not report relevant outcomes, lacked experimental data, or had missing data were excluded.
5. To ensure accuracy and reliability, only studies published in English were included. Studies with English abstracts but non-English full texts were excluded.

### 2.3 Study selection

Two researchers (JP and WR) independently reviewed the retrieved studies using EndNote reference management software. The screening process was conducted independently, with a third researcher (MY) reviewing and confirming the results. Initially, duplicate studies were removed, followed by a thorough examination of titles and abstracts to identify and exclude irrelevant studies. Studies that preliminarily met the inclusion criteria were then fully reviewed for final confirmation. Any discrepancies during the independent evaluation and screening process were resolved through discussion by the research team.

### 2.4 Data extraction

Relevant data from the included studies were independently extracted by two researchers and systematically organized using standardized forms. If study data were presented in graphical form, the data were extracted using specialized software (Engauge Digitizer). In cases of inconsistencies in data extraction, the results were first checked by a third researcher and then discussed by the research team to reach a final decision. Key information was extracted to provide a clear overview of the included studies, including the author, publication year, country or region of the study, participants’ age, sample size, specific intervention methods, details of the exercise intervention (total duration, frequency, and duration of each session), and outcome measures. Additionally, participants were categorized by age group, including children, young adults within the adult population, and adults inclusive of the middle-aged population.

### 2.5 Risk of bias of individual studies

In assessing the risk of bias (ROB), seven domains were considered: random sequence generation, allocation concealment, blinding of participants and personnel, blinding of outcome assessment, incomplete outcome data, selective reporting, and other biases. The results were obtained using the Cochrane risk of bias assessment tool, with the aid of Review Manager 5.4 software. Typically, studies were categorized as low-risk, moderate-risk, or high-risk based on the evaluation of the seven criteria (27).

### 2.6 Data analysis

To clearly present the results, standardized mean differences (SMD) and 95% confidence intervals (CIs) were calculated. Typically, the standardized mean difference (SMD) is categorized into three effect sizes: small (SMD ≤ 0.2), medium (SMD ≤ 0.5), and large (SMD > 0.8) (28). The degree of heterogeneity across studies was assessed using the I² statistic derived from the Cochrane Q test. Heterogeneity levels were classified as low (I² ≤ 25%), moderate (25% < I² ≤ 50%), substantial (50% < I² ≤ 75%), and considerable (I² > 75%) (29). When heterogeneity is low (P ≥ 0.1 and I² ≤ 50%), a fixed-effects model is used for analysis; otherwise, a random-effects model is applied.

To explore potential sources of heterogeneity, a subgroup analysis was conducted based on categorical variables, primarily focusing on different age groups. When the number of studies exceeded 10, funnel plots were constructed to assess potential publication bias and the impact of small sample sizes on outcome measures. Despite the limitations posed by the number of studies, Begg’s and Egger’s tests were performed to assess the presence of significant publication bias. Additionally, sensitivity analysis was conducted by sequentially excluding individual studies. Statistical analyses were conducted using Stata 15.1, with significance set at P < 0.05.

## 3 Results

### 3.1 Study identification and selection

**Figure 1:**
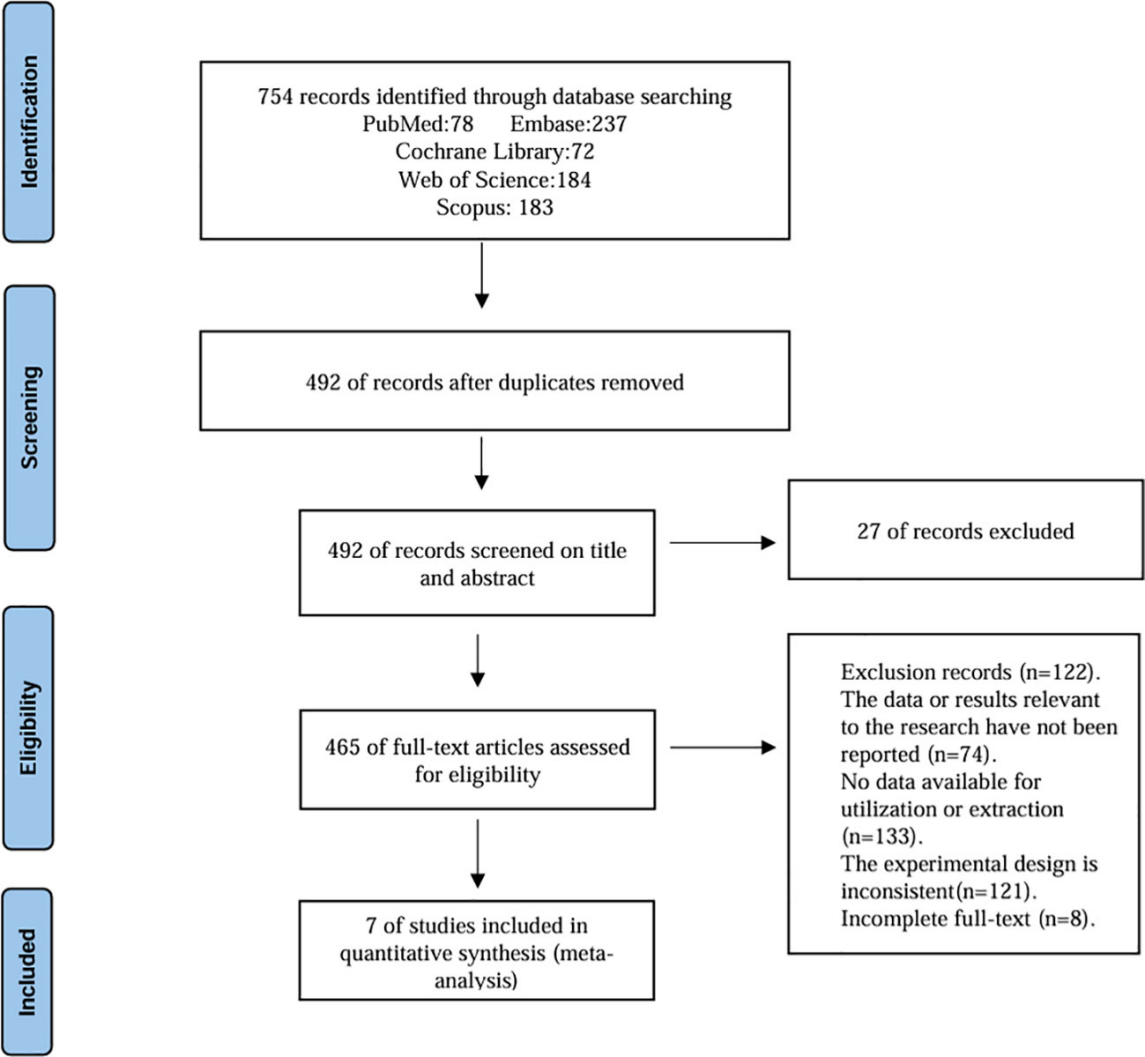
Flowchart of literature search.

Following the initial database search, studies were screened through a stepwise process that involved removing duplicates, reading titles and abstracts, and then reviewing full texts. Ultimately, seven studies were included in the quantitative synthesis. The detailed procedures for each step are illustrated in **Figure 1**.

### 3.2 Study characteristics

The details of the studies included in the quantitative synthesis are presented in **Table 1**. The studies span a time range from 2004 to 2023. They involve participants from five countries or regions: China, the United States, Australia, Iran, and the United Kingdom, with two studies each from China and the United States, and one from each of the remaining countries. The total number of participants across these studies was 268. In two of the studies, participants were administered stimulant-like medications; two studies clearly reported no medication use, while the remaining studies did not specify whether any stimulant medications were used.

**Table 1:** Study characteristics.

Author	Country	Year	Study setting	Intervention implementers	Age mean+SD	Total/male/female	Intervention	Control	Outcome
Chien-Chih Chou.et al.	China	2017	Community	Professional yoga instructor	T:10.71(1.00) C:10.30(1.07)	T:24/19/5 C:25/19/6	Yoga program Length of Intervention: 8 weeks Freq: 2 times a week Duration: 40 min	Normal life	Attention
Pauline. S. Jensen, et al.	Australia	2004	Clinical	Expert	T:10.63(1.78) C:9.35(1.70)	T:11/11/0 C:8/8/0	Yoga program Length of Intervention: 20 weeks Freq: 1 time a week Duration:60min	Cooperative games and activities Freq: 1 time one month Duration:60min	Attention; Executive Functioning; Hyperactive/Impulsive; Emotion
Kathryn Fritz, et al.	United States	2022	Community	Professional yoga instructor	20.16(1.46)	T:11/0/11 C:16/0/16	Bikram yoga training Length of Intervention: 6 weeks Freq: 2 times a week Duration: 90 min	Wait-list control group.	Attention; Executive Functioning; Emotion
Sheng Wang	China	2023	No Report	No Report	T:4.3(0.58)C:3.8(0.83)	T:12/8/4 C:11/7/4	Yoga Length of Intervention: 8 weeks Freq: 3 times a week Duration: 20 min	Not receiving any treatment	Emotion
Kouhbanani, Sakineh Soltani, et al.	Iran	2022	Community	Professional sports coach	T:35.24(11.49) C:35.40(11.01)	T:25/0/25 C:27/0/27	Pilates Length of Intervention: 24 weeks Freq: 3 times a week Duration: 45 min	Maintain previous treatment	Attention; Executive Functioning
Alexander K. Converse, et al.	United States	2020	Community	Professional sports coach	20.7 ± 1.5	T:9/4/5 C:7/2/5	Tai Chi Length of Intervention: 7 weeks Freq: 2 times a week Duration: 60 min	No training received	Attention; Executive Functioning; Hyperactive/Impulsive
Larisa M. Dinu, et al.	United Kingdom	2023	Community	Video Learning	18-35	No Report	Hatha yoga Duration: 12 min	Aerobic cycling Duration: 10 min	Attention; Executive Functioning; Hyperactive/Impulsive

The types of MBE interventions included yoga, Pilates, and Tai Chi, with yoga being the focus of five studies. Among the interventions, six studies were considered long-term interventions, while only one study was classified as an acute intervention, which consisted of a 12-minute session including warm-up activities. Additionally, if a study reported multiple outcome measures related to a single outcome, we extracted data from up to two measures for quantitative synthesis. The outcome measures included attention, executive function, emotional regulation, and hyperactivity/impulsivity.

### 3.3 Risk of bias

**Figure 2:**
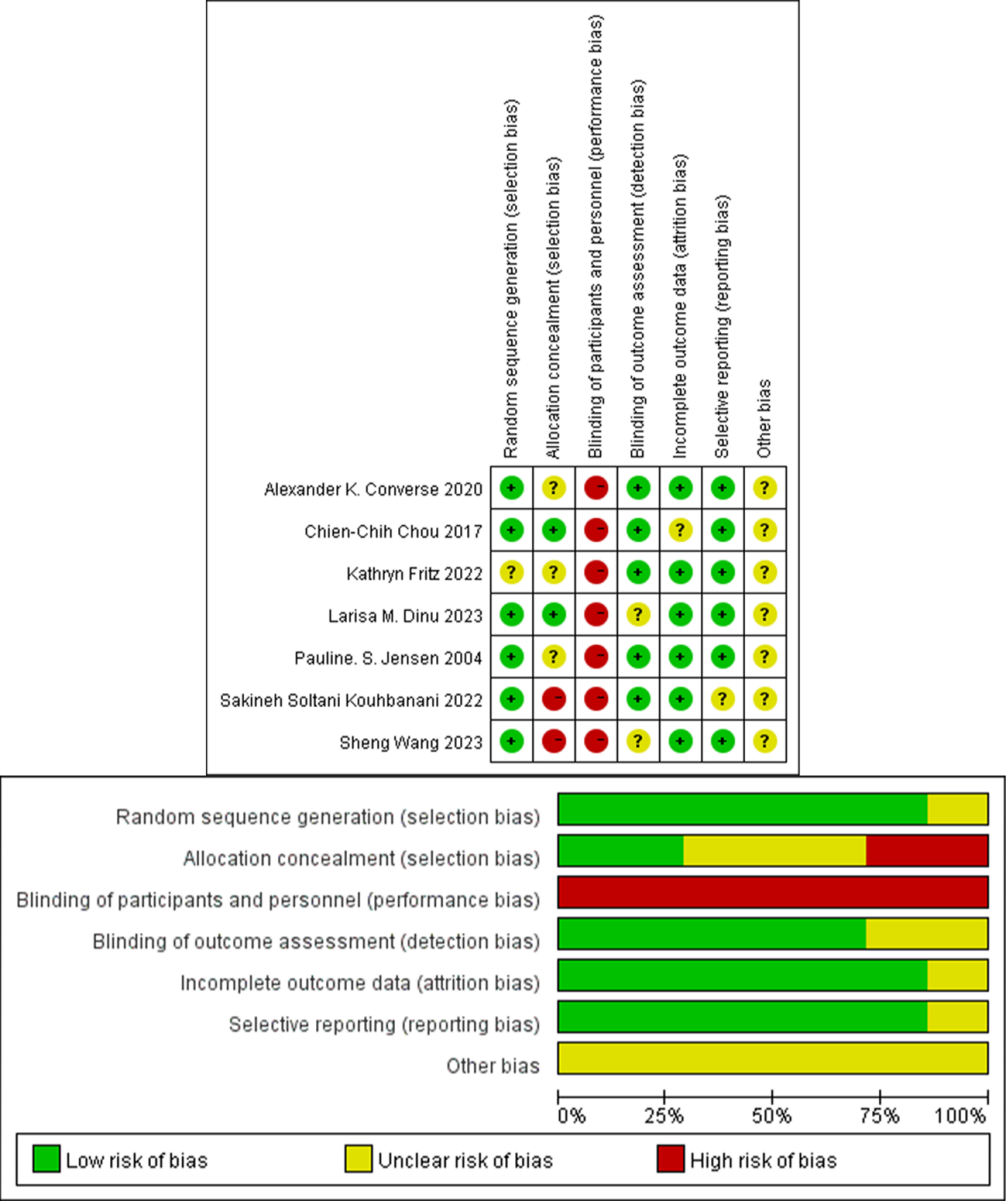
Risk of publication bias.

Since the interventions involved physical activities, it was generally evident to the personnel administering the interventions which group they were working with, Consequently, high risk was generally assigned during the assessment of performance bias. Moreover, selection bias was also deemed high risk in two studies. Overall, five studies were classified as moderate risk, and two as high risk. The detailed risk assessment results are presented in **Figure 2**.

### 3.4 Meta-analysis

#### 3.4.1 The effect of MBE on attention

**Figure 3:**
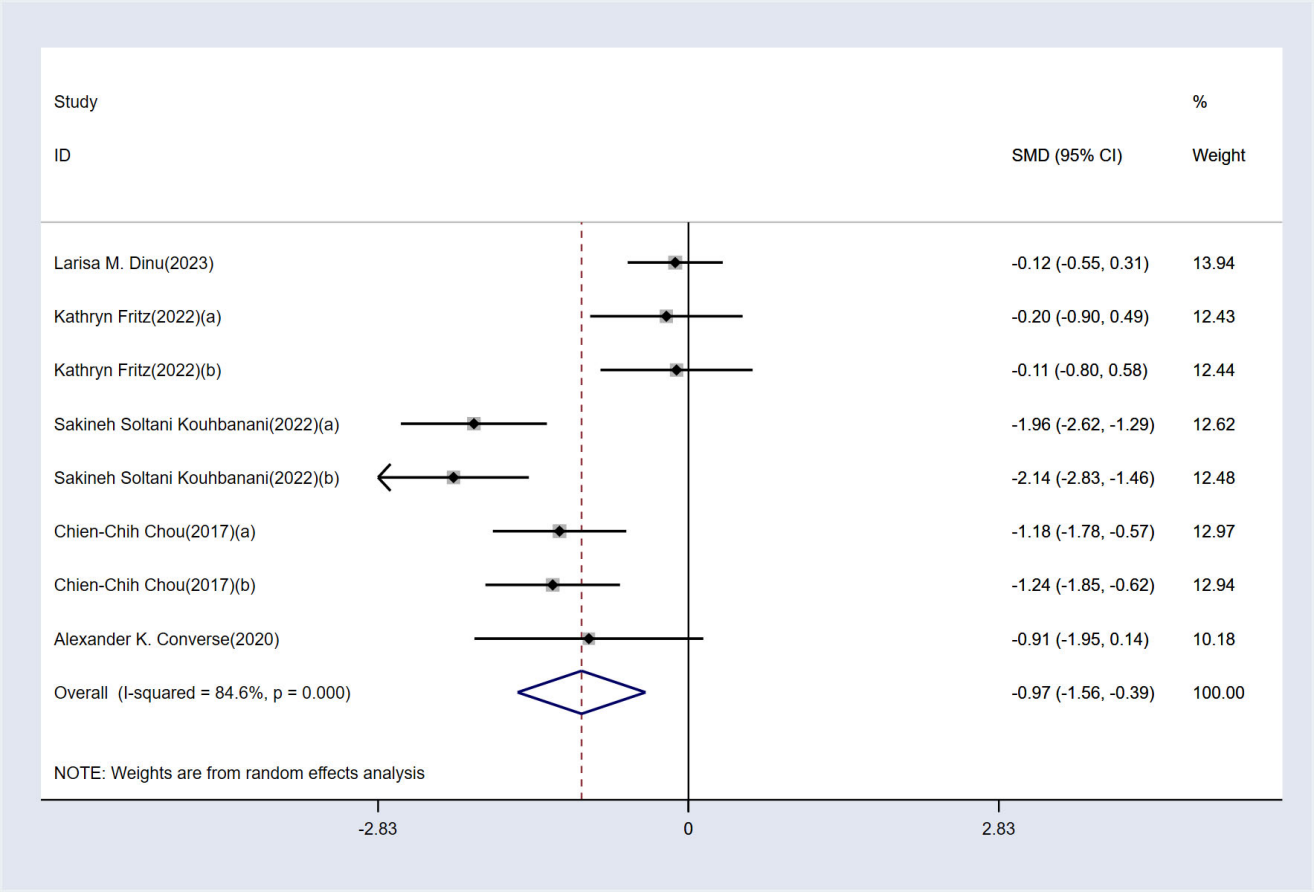
Meta-analysis results.

The analysis revealed high heterogeneity for this outcome (I² = 84.5%, P < 0.1). Consequently, a random-effects model was employed for the analysis. The results indicate that, compared to the control group, MBE significantly improved attention in ADHD patients [SMD = -0.97, 95% CI (-1.56, -0.39), P < 0.05]. The detailed results are presented in **Figure 3**.

#### 3.4.2 The effect of MBE on executive function, emotional problems, and hyperactivity/impulsivity

**Figure 4:**
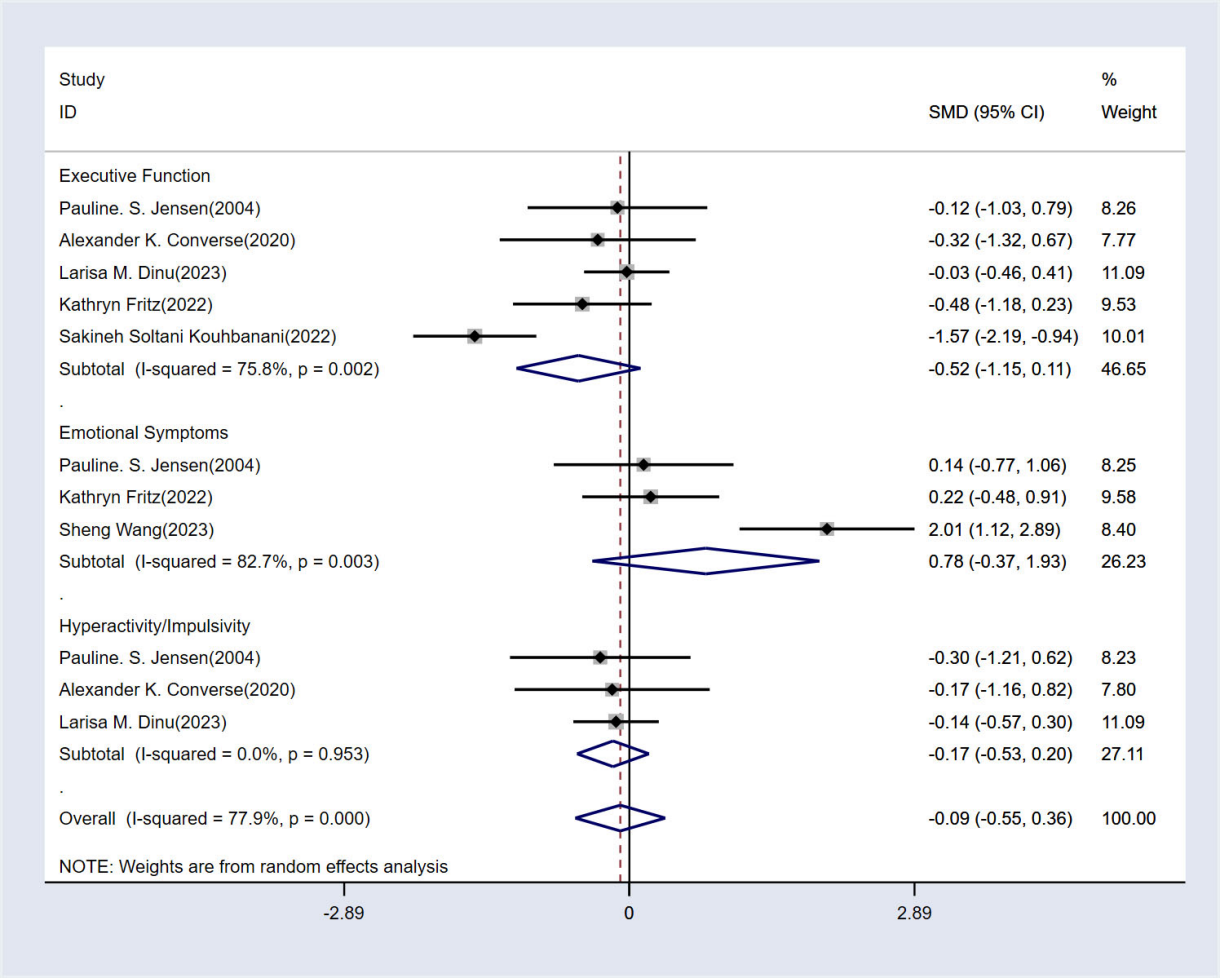
Analysis chart of the other three indicators.

The results are displayed in **Figure 4**. The analysis shows that MBE did not have a significant impact on executive function [SMD = -0.52, 95% CI (-1.15, 0.11), P > 0.05], emotional symptoms [SMD = 0.78, 95% CI (-0.37, 1.93), P > 0.05], or hyperactivity/impulsivity [SMD = -0.17, 95% CI (-0.53, 0.2), P > 0.05]. Therefore, MBE cannot be considered an effective treatment for these symptoms.

#### 3.4.3 Subgroup analysis

**Figure 5:**
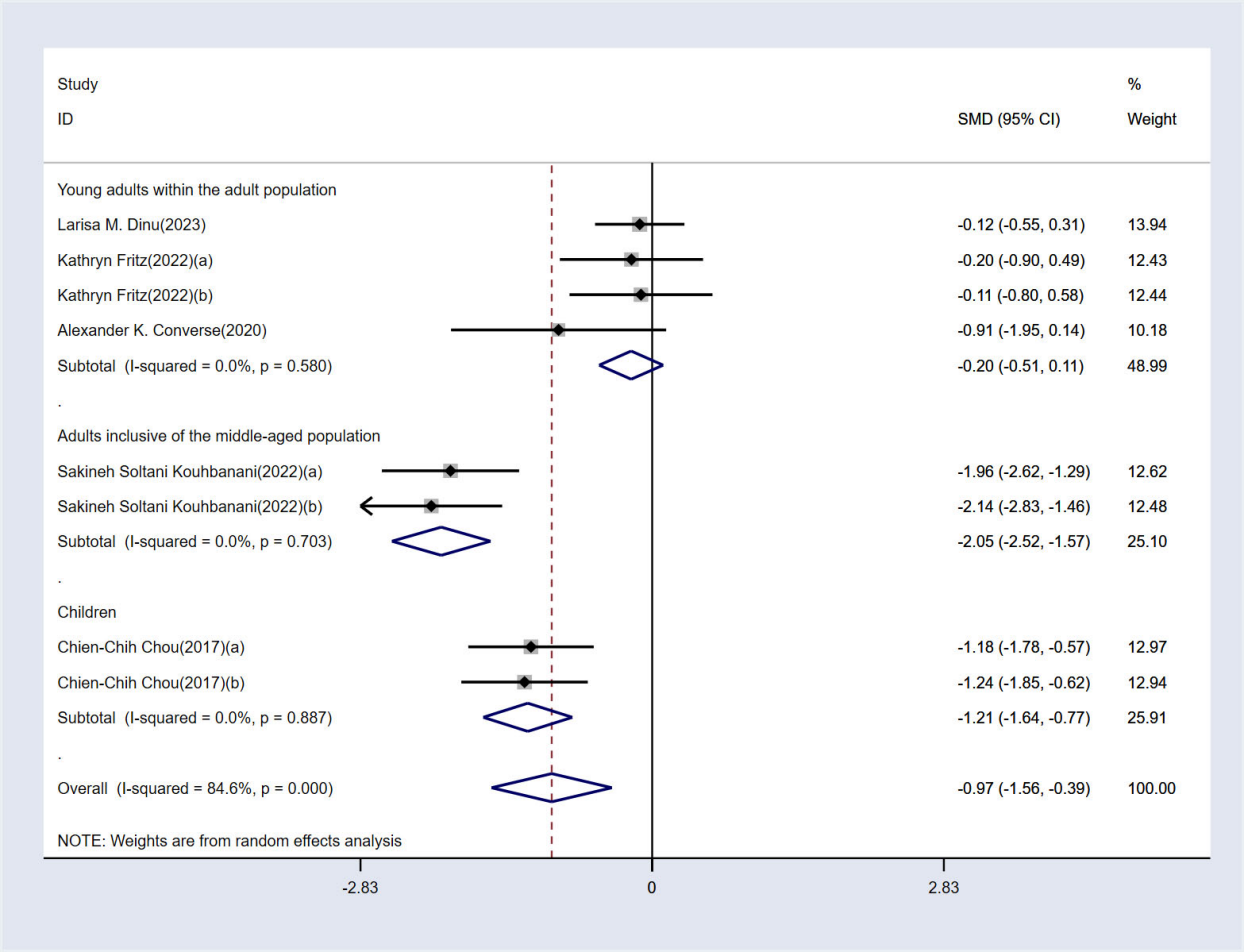
Subgroup analysis results.

A subgroup analysis based on age was conducted to identify potential sources of heterogeneity in the attention outcome. The results of the subgroup analysis are shown in **Figure 5**. No significant differences were found between the three age groups (I² < 50%, P > 0.1 for all groups). Generally, if the within-group differences are insignificant and the between-group differences are significant, it can be inferred that the subgroup classification is a source of heterogeneity. Additionally, MBE was found to be effective in improving attention in children and middle-aged adults but was ineffective in young adults.

### 3.5 Publication bias test

Visual inspection of the funnel plot suggested a potential publication bias due to noticeable asymmetry. However, sensitivity analysis, performed by sequentially excluding individual studies, did not reveal any study with a significant impact on the overall results. Moreover, the results of Egger’s test provided no evidence of significant publication bias (P = 0.757 > 0.05). Therefore, our findings can be considered robust. The funnel plot and sensitivity analysis figures are provided in **Supplementary Material 2**.

## 4 Discussion

To date, this study is the first to employ meta-analysis to investigate the effects of MBE on patients with ADHD. The results of the meta-analysis, based on seven included studies, indicate that MBE can significantly improve attention in ADHD patients compared to control groups.

Previous research has demonstrated that physical exercise can enhance attention in individuals with ADHD (30, 31). Since MBE is a form of physical exercise, it is highly likely that it shares similar benefits. From the perspective of movement patterns, MBE generally involves tasks that require attention control. For instance, practicing yoga demands that participants consciously maintain specific postures for a certain duration, thereby requiring sustained attention. Pilates emphasizes concentration and the precise control of movements, with a focus on the body being directed by the mind (32). Dance involves rapid transitions between various movements (33, 34), necessitating a high degree of attentional focus and quick, accurate responses (35). Similarly, other MBE forms, such as Tai Chi and Qigong, also involve tasks that require attention control (36, 37). Additionally, the focused control and expansive movement characteristics of MBE may temporarily modulate hyperactive brain activity in children practicing MBE, thus potentially enhancing attention. Overall, the nature of MBE itself directly correlates with improvements in attention.

The mechanism underlying MBE’s ability to enhance attention may be akin to that of physical activity. As a form of physical activity, MBE can be viewed as a neuroenhancer, improving cognitive abilities through acute and long-term effects on monoaminergic neurotransmission, neurotrophic signaling, and neuroplasticity (38). Additionally, metabolic byproducts and myokines released during muscle contractions in MBE can enhance brain function, including attention (39). Patients with ADHD often exhibit heightened levels of stress responses, which may further exacerbate attention deficits. Mindfulness-based exercise (MBE), characterized by relaxation techniques such as breathing and meditation, has the potential to reduce stress hormone levels, thereby alleviating attention problems induced by stress (40). In adults with ADHD, MBE interventions enhance self-regulation abilities through breathing control, bodily awareness, and emotional balance. Regular practice may strengthen self-management skills, leading to improved concentration. Overall, for both children and adults, this exercise, whether directly or indirectly, contributes to the overall improvement in patients’ physical health and brain plasticity, thereby alleviating attention-related symptoms. Although various mechanisms may underlie these improvements, definitive explanations have yet to be established.

Compared to other methods, the unique benefits of MBE (Mind-Body Exercise) for ADHD patients are particularly significant in childhood. During this critical period for cognitive and emotional regulation development (41), MBE, through its distinct mindfulness practices and focus training, effectively helps children with ADHD build a solid foundation for self-regulation. Additionally, children are more susceptible to the side effects of medications, particularly those affecting the psychological and nervous systems (42, 43). MBE, as a non-pharmacological intervention, has the advantage of no notable side effects, and its low cost makes it more suitable for the long-term management of children with ADHD, especially for families facing financial constraints. Unlike medications that directly target neurotransmitters, MBE works by gradually cultivating intrinsic focus, aiming to produce long-lasting positive effects in various aspects of daily life and academic performance for children with ADHD. Overall, the application of MBE in childhood may benefit the physical and mental development of ADHD patients and progressively mitigate the adverse effects associated with ADHD.

However, our study did not find evidence that MBE improves executive function, emotional regulation, or hyperactivity/impulsivity in ADHD patients. There is evidence suggesting that deficits in executive function may be a defining feature of ADHD (44). Expecting a single intervention like MBE to ameliorate such a fundamental aspect of the disorder may be overly optimistic. In clinical practice, stimulant medications are typically used to improve executive function in ADHD, with research showing that methylphenidate positively affects response inhibition and working memory (45). Exploring non-pharmacological or side-effect-free treatments that could potentially enhance executive function in ADHD patients may represent a new avenue for future research.

Regarding the lack of significant effects on emotional regulation, it is plausible that MBE interventions do not adequately address tasks related to emotional control, and thus may not exert a substantial impact in this area. Lastly, concerning hyperactivity and impulsivity, it is important to consider the developmental mechanisms in adolescent ADHD patients. Adolescents with higher levels of hyperactivity/impulsivity tend to exhibit slower cortical thinning, particularly in areas such as the prefrontal cortex, bilateral precentral/middle frontal gyri, extending inferiorly to the medial wall of the frontal lobe and the anterior cingulate gyrus, as well as the orbitofrontal cortex and right inferior frontal gyrus (46). Since MBE improves certain symptoms of ADHD through the combined effects of physical and mental engagement, it may not be capable of altering the physical aspects of developmental processes.

This study has several strengths but also limitations. Our research provides preliminary meta-analytic evidence for the efficacy of MBE in treating ADHD patients, and the inclusion of randomized controlled trials in the quantitative synthesis enhances the stability and reliability of the findings. However, there are limitations that may impact the interpretation of the results. The number of studies included was relatively small, which may limit the strength of the conclusions. Additionally, subgroup analyses were constrained by the limited number of studies, potentially affecting the results. Furthermore, most of the included studies did not explicitly differentiate between genders, preventing an examination of whether gender differences may influence the effects of MBE. Future research should aim to include a larger number of studies and more RCTs to provide higher levels of evidence. Additionally, investigating whether MBE has different effects on ADHD patients of varying ages would be valuable.

## 5 Conclusion

Research indicates that MBE can improve attention symptoms in ADHD patients, though it does not significantly impact executive function or emotional symptoms. The focus-oriented characteristics of MBE likely contribute to its effectiveness in enhancing attention. However, MBE alone may be insufficient to activate brain regions associated with complex executive functions, thus showing limited efficacy in that area. Consequently, further studies are needed to provide additional evidence.

## Data Availability

The original contributions presented in the study are included in the article/**Supplementary Material**. Further inquiries can be directed to the corresponding author.
