# Supplementary figures and images for: Effects of mind-body exercise on individuals with ADHD: a systematic review and meta-analysis
Source: Front Psychiatry. 2024 Dec 9;15:1490708. doi: 10.3389/fpsyt.2024.1490708 (PMC11663905; doi:10.3389/fpsyt.2024.1490708)

Supplementary Material


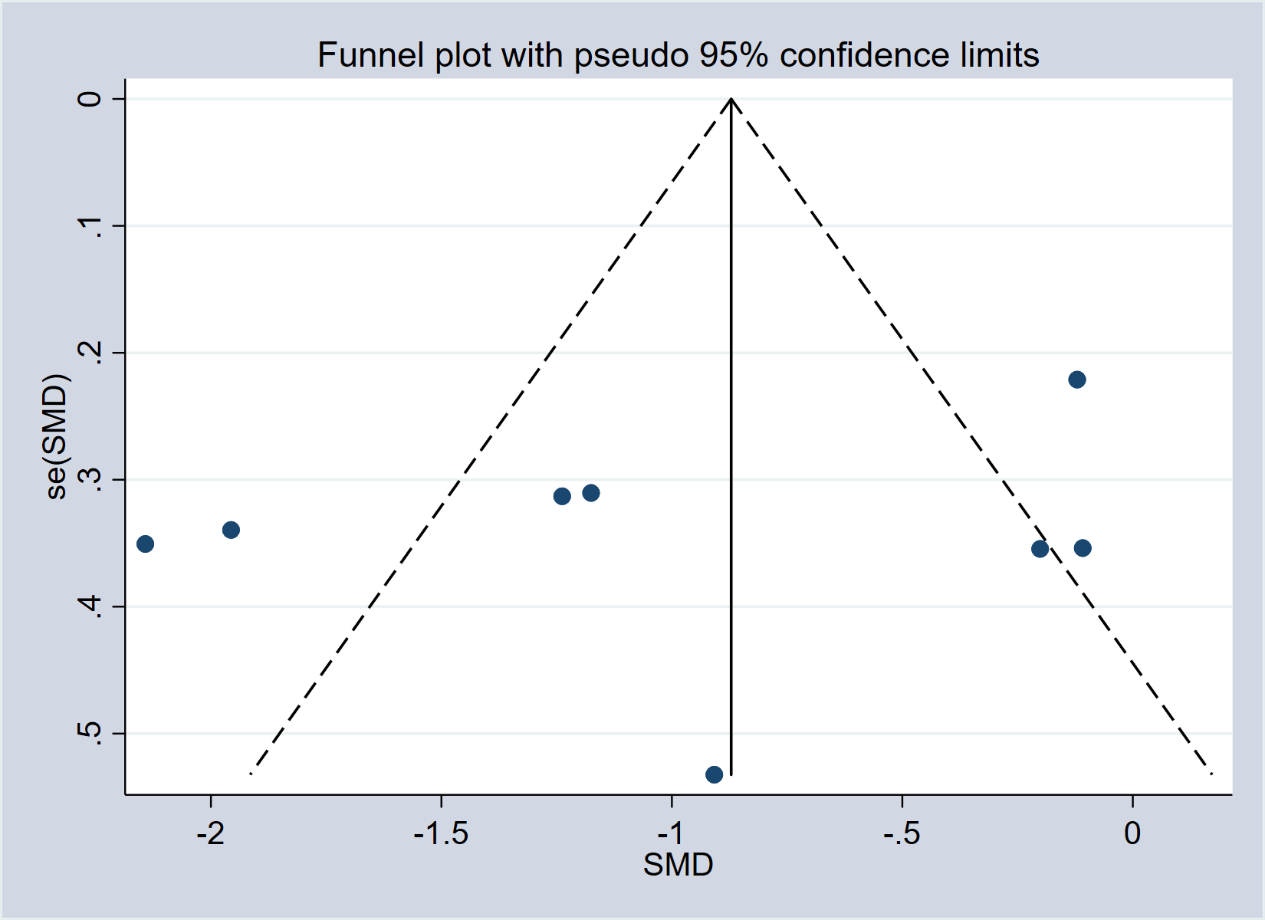


Funnel chart

Sensitivity analysis chart

Supplement: Supplementary file 1 [file DataSheet1.docx]
