# Supplementary material for: Effects of mind-body exercise on individuals with ADHD: a systematic review and meta-analysis
Source: Front Psychiatry. 2024 Dec 9;15:1490708. doi: 10.3389/fpsyt.2024.1490708 (PMC11663905; doi:10.3389/fpsyt.2024.1490708)
Supplement: Supplementary file 2 [file Table1.docx]

Supplementary Material

**Detailed Literature Search Strategy**

**Appendix 1** Search Strategy

| Database | Search strategy | amount |
| --- | --- | --- |
| PubMed | | |
| #1 | Search: ADHD[MeSH Terms] | 36161 |
| #2 | Search: ((((((((((((((((((((ADDH) OR (Attention Deficit Disorders with Hyperactivity)) OR (Attention Deficit Hyperactivity Disorders)) OR (Attention Deficit Hyperactivity Disorder)) OR (Attention Deficit-Hyperactivity Disorder)) OR (Attention Deficit-Hyperactivity Disorders)) OR (Deficit-Hyperactivity Disorder, Attention)) OR (Deficit-Hyperactivity Disorders, Attention)) OR (Disorder, Attention Deficit-Hyperactivity)) OR (Disorders, Attention Deficit-Hyperactivity)) OR (Hyperkinetic Syndrome)) OR (Syndromes, Hyperkinetic)) OR (Attention Deficit Disorder)) OR (Attention Deficit Disorders)) OR (Deficit Disorder, Attention)) OR (Deficit Disorders, Attention)) OR (Disorder, Attention Deficit)) OR (Disorders, Attention Deficit)) OR (Brain Dysfunction, Minimal)) OR (Dysfunction, Minimal Brain)) OR (Minimal Brain Dysfunction) | 55478 |
| #3 | #1 OR #2 | 55478 |
| #4 | Search: Mind Body Exercise[MeSH Terms] | 803 |
| #5 | Search: ((((((((((((((((((((((((((((((Mind Body Exercise) OR (Mind-body exercise)) OR (Exercise, Mind-Body)) OR (Tai Ji Quan)) OR (Taiji)) OR (Taijiquan)) OR (Yoga)) OR (Qi Gong)) OR (Nei Yang Gong)) OR (Dance)) OR (Ballet)) OR (Square Dance)) OR (Dance, Square)) OR (Hip-Hop Dance)) OR (Dance, Hip-Hop)) OR (Hip Hop Dance)) OR (Jazz Dance)) OR (Dance, Jazz)) OR (Tap Dance)) OR (Dance, Tap)) OR (Modern Dance)) OR (Dance, Modern)) OR (Salsa Dancing)) OR (Dancing, Salsa)) OR (Line Dancing)) OR (Dancing, Line)) OR (Pilates-Based Exercises)) OR (Exercises, Pilates-Based)) OR (Pilates Based Exercises)) OR (Pilates Training)) OR (Training, Pilates) | 33258 |
| #6 | #4 OR #5 | 33258 |
| #7 | #3 AND #6 | 78 |

| **Database** | **Search strategy** | **amount** |
| --- | --- | --- |
| **Embase** | | |
| #1 | 'adhd'/exp OR adhd OR 'addh' OR (attention AND deficit AND disorders AND with AND hyperactivity) OR (attention AND deficit AND hyperactivity AND disorders) OR (attention AND deficit AND hyperactivity AND disorder) OR (attention AND 'deficit hyperactivity' AND disorder) OR (attention AND 'deficit hyperactivity' AND disorders) OR ('deficit hyperactivity' AND disorder, AND attention) OR ('deficit hyperactivity' AND disorders, AND attention) OR (disorder, AND attention AND 'deficit hyperactivity') OR (hyperkinetic AND syndrome) OR (syndromes, AND hyperkinetic) OR (attention AND deficit AND disorder) OR (attention AND deficit AND disorders) OR (deficit AND disorder, AND attention) OR (deficit AND disorders, AND attention) OR (disorder, AND attention AND deficit) OR (disorders, AND attention AND deficit) OR (brain AND dysfunction, AND minimal) OR (dysfunction, AND minimal AND brain) OR (minimal AND brain AND dysfunction) | 99036 |
| #2 | 'mind body exercise'/exp OR 'mind body exercise' OR (('mind'/exp OR mind) AND ('body'/exp OR body) AND ('exercise'/exp OR exercise)) OR ('mind body' AND exercise) OR (exercise, AND 'mind body') OR (tai AND ji AND quan) OR taiji OR taijiquan OR yoga OR (qi AND gong) OR (nei AND yang AND gong) OR dance OR ballet OR (square AND dance) OR (dance, AND square) OR ('hip hop' AND dance) OR (dance, AND 'hip hop') OR (hip AND hop AND dance) OR (jazz AND dance) OR (dance, AND jazz) OR (tap AND dance) OR (dance, AND tap) OR (modern AND dance) OR (dance, AND modern) OR (salsa AND dancing) OR (dancing, AND salsa) OR (line AND dancing) OR (dancing, AND line) OR ('pilates based' AND exercises) OR (exercises, AND 'pilates based') OR (pilates AND based AND exercises) OR (pilates AND training) OR (training, AND pilates) | 42203 |
| #3 | #1 AND #2 | 237 |

| **Database** | **Search strategy** | **amount** |
| --- | --- | --- |
| **Web of Science** | | |
| #1 | (((((((((((((((((((((TS=(ADHD)) OR TS=(ADDH)) OR TS=(Attention Deficit Disorders with Hyperactivity)) OR TS=(Attention Deficit Hyperactivity Disorders)) OR TS=(Attention Deficit Hyperactivity Disorder)) OR TS=(Attention Deficit-Hyperactivity Disorder)) OR TS=(Attention Deficit-Hyperactivity Disorders)) OR TS=(Deficit-Hyperactivity Disorder, Attention)) OR TS=(Deficit-Hyperactivity Disorders, Attention)) OR TS=(Disorder, Attention Deficit-Hyperactivity)) OR TS=(Disorders, Attention Deficit-Hyperactivity)) OR TS=(Hyperkinetic Syndrome)) OR TS=(Syndromes, Hyperkinetic)) OR TS=(Attention Deficit Disorder)) OR TS=(Attention Deficit Disorders)) OR TS=(Deficit Disorder, Attention)) OR TS=(Deficit Disorders, Attention)) OR TS=(Disorder, Attention Deficit)) OR TS=(Disorders, Attention Deficit)) OR TS=(Brain Dysfunction, Minimal)) OR TS=(Dysfunction, Minimal Brain)) OR TS=(Minimal Brain Dysfunction) and Preprint Citation Index (Exclude – Database) | 111579 |
| #2 | (((((((((((((((TS=(Mind Body Exercise)) OR TS=(Mind-body exercise)) OR TS=(Exercise, Mind-Body)) OR TS=(Tai Ji Quan)) OR TS=(Taiji)) OR TS=(Taijiquan)) OR TS=(Yoga)) OR TS=(Qi Gong)) OR TS=(Nei Yang Gong)) OR TS=(Dance)) OR TS=(Ballet)) OR TS=(Square Dance)) OR TS=(Dance, Square)) OR TS=(Hip-Hop Dance)) OR TS=(Dance, Hip-Hop)) OR TS=(Hip Hop Dance) and Preprint Citation Index (Exclude – Database) | 65537 |
| #3 | ((((((((((((((TS=(Jazz Dance)) OR TS=(Dance, Jazz)) OR TS=(Tap Dance)) OR TS=(Dance, Tap)) OR TS=(Modern Dance)) OR TS=(Dance, Modern)) OR TS=(Salsa Dancing)) OR TS=(Dancing, Salsa)) OR TS=(Line Dancing)) OR TS=(Dancing, Line)) OR TS=(Pilates-Based Exercises)) OR TS=(Exercises, Pilates-Based)) OR TS=(Pilates Based Exercises)) OR TS=(Pilates Training)) OR TS=(Training, Pilates) and Preprint Citation Index (Exclude – Database) | 7179 |
| #4 | #2 or #3 | 66319 |
| #5 | #1 and #4 | 184 |

| Database | Search strategy | amount |
| --- | --- | --- |
| Cochrane | | |
| #1 | (ADHD):ti,ab,kw OR (ADDH):ti,ab,kw OR (Attention Deficit Disorders with Hyperactivity):ti,ab,kw OR (Attention Deficit Hyperactivity Disorders):ti,ab,kw OR (Attention Deficit Hyperactivity Disorder):ti,ab,kw | 7682 |
| #2 | (Attention Deficit-Hyperactivity Disorder):ti,ab,kw OR (Attention Deficit-Hyperactivity Disorders):ti,ab,kw OR (Deficit-Hyperactivity Disorder, Attention):ti,ab,kw OR (Deficit-Hyperactivity Disorders, Attention):ti,ab,kw OR (Disorder, Attention Deficit-Hyperactivity):ti,ab,kw | 5726 |
| #3 | (Disorders, Attention Deficit-Hyperactivity):ti,ab,kw OR (Hyperkinetic Syndrome):ti,ab,kw OR (Syndromes, Hyperkinetic):ti,ab,kw OR (Attention Deficit Disorder):ti,ab,kw OR (Attention Deficit Disorders):ti,ab,kw | 8457 |
| #4 | (Deficit Disorder, Attention):ti,ab,kw OR (Deficit Disorders, Attention):ti,ab,kw OR (Disorder, Attention Deficit):ti,ab,kw OR (Disorders, Attention Deficit):ti,ab,kw | 8399 |
| #5 | (Brain Dysfunction, Minimal):ti,ab,kw OR (Dysfunction, Minimal Brain):ti,ab,kw OR (Minimal Brain Dysfunction):ti,ab,kw | 316 |
| #6 | #1 or #2 or #3 or #4 or #5 | 9427 |
| #7 | (Mind Body Exercise):ti,ab,kw OR (Mind-body exercise):ti,ab,kw OR (Exercise, Mind-Body):ti,ab,kw OR (Tai Ji Quan):ti,ab,kw OR (Taiji):ti,ab,kw | 1490 |
| #8 | (Taijiquan):ti,ab,kw OR (Yoga):ti,ab,kw OR (Qi Gong):ti,ab,kw OR (Nei Yang Gong):ti,ab,kw OR (Dance):ti,ab,kw | 7624 |
| #9 | (Ballet):ti,ab,kw OR (Square Dance):ti,ab,kw OR (Dance, Square):ti,ab,kw OR (Hip-Hop Dance):ti,ab,kw OR (Dance, Hip-Hop):ti,ab,kw | 194 |
| #10 | (Hip Hop Dance):ti,ab,kw OR (Jazz Dance):ti,ab,kw OR (Dance, Jazz):ti,ab,kw OR (Tap Dance):ti,ab,kw OR (Dance, Tap):ti,ab,kw | 43 |
| #11 | (Modern Dance):ti,ab,kw OR (Dance, Modern):ti,ab,kw OR (Salsa Dancing):ti,ab,kw OR (Dancing, Salsa):ti,ab,kw OR (Line Dancing):ti,ab,kw | 99 |
| #12 | (Dancing, Line):ti,ab,kw OR (Pilates-Based Exercises):ti,ab,kw OR (Exercises, Pilates-Based):ti,ab,kw | 142 |
| #13 | (Pilates Based Exercises):ti,ab,kw OR (Pilates Training):ti,ab,kw OR (Training, Pilates):ti,ab,kw | 739 |
| #14 | #7 or #8 or #9 or #10 or #11 or #12 or #13 | 9496 |
| #15 | #6 and #14 | 72 |

| Database | Search strategy | amount |
| --- | --- | --- |
| Scopus | | |
| #1 | ( TITLE-ABS-KEY ( "adhd" ) OR TITLE-ABS-KEY ( "addh" ) OR TITLE-ABS-KEY ( "attention deficit disorders with hyperactivity" ) OR TITLE-ABS-KEY ( "attention deficit hyperactivity disorders" ) OR TITLE-ABS-KEY ( "attention deficit hyperactivity disorder" ) OR TITLE-ABS-KEY ( "attention deficit-hyperactivity disorder" ) OR TITLE-ABS-KEY ( "attention deficit-hyperactivity disorders" ) OR TITLE-ABS-KEY ( "deficit-hyperactivity disorder, attention" ) OR TITLE-ABS-KEY ( "deficit-hyperactivity disorders, attention" ) OR TITLE-ABS-KEY ( "disorder, attention deficit-hyperactivity" ) OR TITLE-ABS-KEY ( "disorders, attention deficit-hyperactivity" ) OR TITLE-ABS-KEY ( "hyperkinetic syndrome" ) OR TITLE-ABS-KEY ( "syndromes, hyperkinetic" ) OR TITLE-ABS-KEY ( "attention deficit disorder" ) OR TITLE-ABS-KEY ( "attention deficit disorders" ) OR TITLE-ABS-KEY ( "deficit disorder, attention" ) OR TITLE-ABS-KEY ( "deficit disorders, attention" ) OR TITLE-ABS-KEY ( "disorder, attention deficit" ) OR TITLE-ABS-KEY ( "disorders, attention deficit" ) OR TITLE-ABS-KEY ( "brain dysfunction, minimal" ) OR TITLE-ABS-KEY ( "dysfunction, minimal brain" ) OR TITLE-ABS-KEY ( "minimal brain dysfunction" ) ) | 88089 |
| #2 | ( TITLE-ABS-KEY ( "mind body exercise" ) OR TITLE-ABS-KEY ( "mind-body exercise" ) OR TITLE-ABS-KEY ( "exercise, mind-body" ) OR TITLE-ABS-KEY ( "tai ji quan" ) OR TITLE-ABS-KEY ( "taiji" ) OR TITLE-ABS-KEY ( "taijiquan" ) OR TITLE-ABS-KEY ( "yoga" ) OR TITLE-ABS-KEY ( "qi gong" ) OR TITLE-ABS-KEY ( "nei yang gong" ) OR TITLE-ABS-KEY ( "dance" ) OR TITLE-ABS-KEY ( "ballet" ) OR TITLE-ABS-KEY ( "square dance" ) OR TITLE-ABS-KEY ( "dance, square" ) OR TITLE-ABS-KEY ( "hip-hop dance" ) OR TITLE-ABS-KEY ( "dance, hip-hop" ) OR TITLE-ABS-KEY ( "hip hop dance" ) OR TITLE-ABS-KEY ( "jazz dance" ) OR TITLE-ABS-KEY ( "dance, jazz" ) OR TITLE-ABS-KEY ( "tap dance" ) OR TITLE-ABS-KEY ( "dance, tap" ) OR TITLE-ABS-KEY ( "modern dance" ) OR TITLE-ABS-KEY ( "dance, modern" ) OR TITLE-ABS-KEY ( "salsa dancing" ) OR TITLE-ABS-KEY ( "dancing, salsa" ) OR TITLE-ABS-KEY ( "line dancing" ) OR TITLE-ABS-KEY ( "dancing, line" ) OR TITLE-ABS-KEY ( "pilates-based exercises" ) OR TITLE-ABS-KEY ( "exercises, pilates-based" ) OR TITLE-ABS-KEY ( "pilates based exercises" ) OR TITLE-ABS-KEY ( "pilates training" ) OR TITLE-ABS-KEY ( "training, pilates" ) ) | 61684 |
| #3 | #1 AND #2 | 183 |
